# Pulpal response following SDF application for deep carious lesions:An *in vivo *study

**DOI:** 10.6026/973206300223491

**Published:** 2026-06-30

**Authors:** Shelly Singh, Sugandha Sharma, Shraddha Shetti, Ashtha Arya, Gaurav Mishra, Sudha Patil

**Affiliations:** 1 Department of Dentistry , School of dental medicine , Anschutz Medical campus , University of Colorado,Aurora, Colorado, USA; 2 Department of Pedodontics, Lather Dental Clinic and Implant Centre, Bhiwani, India; 3 Department of Orthodontics, Bharati Vidyapeeth (Deemed to be University, Pune) Dental College and Hospital, Sangli, Maharashtra, India; 4 Department of Conservative Dentistry and Endodontics, SGT Dental College, Hospital and Research Institute, SGT University, Gurugram, Haryana, India; 5 Department of Pedodontics, College Modern Dental College and Research center Place, Indore, India; 6 Department of Pedodontics, ACPM Dental college and Hospital, Sakri Road, Dhule, Maharashtra, India

**Keywords:** Silver diamine fluoride (SDF), deep carious lesions, pulpal response, primary molars, permanent molars, vital pulp therapy, minimally invasive dentistry

## Abstract

Deep carious lesions often threaten pulpal vitality and limited evidence exists regarding the pulpal response to 38% silver diamine fluoride (SDF) in primary and permanent molars. Therefore, it is of interest to evaluate the pulpal response to 38% SDF in deep carious lesions of primary and permanent molars. Hence, a total of 70 molars (35 primary, 35 permanent) were treated with SDF following selective caries removal and monitored clinically and radiographically for 12 months. Most teeth maintained pulpal vitality, with overall success rates of 80% in primary molars and 85.7% in permanent molars. Failures were mainly associated with spontaneous pain or radiographic periapical changes during follow-up. Thus, we show that SDF is a minimally invasive and biologically compatible option for managing deep caries while preserving pulp vitality in both dentitions.

## Background:

Dental caries remains one of the most prevalent chronic diseases affecting children and adults worldwide. The progression of untreated caries can lead to pulpal inflammation, pain and eventual tooth loss if not managed appropriately [1]. Management of deep carious lesions aims to preserve pulp vitality while arresting disease progression. Contemporary minimally invasive dentistry emphasizes biological approaches that maintain tooth structure and pulpal health [2]. Silver diamine fluoride (SDF) has emerged as a promising non-invasive agent for arresting dental caries. It possesses strong antimicrobial properties and promotes remineralization of demineralized dental tissues [3]. SDF application has been widely studied in primary teeth due to its ability to halt caries progression and reduce the need for invasive restorative procedures. This approach is particularly useful in young or uncooperative patients [4]. The mechanism of SDF involves the combined action of silver ions and fluoride ions. Silver acts as an antibacterial agent, while fluoride supports remineralization and enhances resistance to further demineralization [5]. Previous studies have demonstrated that SDF can significantly inhibit cariogenic bacteria and stabilize carious dentin. These effects contribute to arresting lesion progression and preserving tooth integrity [6]. Recent investigations have also explored the effect of SDF on dentin structure and the dentin-pulp complex. Evidence suggests that it may influence dentinal tubules and potentially protect underlying pulp tissue [7]. Despite the increasing use of SDF in clinical practice, concerns remain regarding its impact on pulpal tissues, especially when applied to deep carious lesions close to the pulp. Understanding the pulpal response is essential for ensuring its safe clinical application [8]. Therefore, evaluating the pulpal response following SDF application in deep carious lesions of both primary and permanent molars is important. Such assessment helps determine its biological compatibility and therapeutic potential [9]. Therefore, it is of interest to assess the pulpal response to SDF application in deep carious lesions of primary and permanent molars.

## Methodology:

The *in vivo* study was carried out in the Department of Pedodontics and Preventive Dentistry and the Department of Conservative Dentistry and Endodontics following ethical approval from the institutional review board and informed consent from all participants or their parents/guardians. The study aimed to assess the pulpal response to the application of 38% silver diamine fluoride (SDF) in deep carious lesions of primary and permanent molars. Selected were deep carious teeth that required conservative management and adhered to stringent vital pulp criteria. The study comprised systemically healthy patients exhibiting deep dentinal caries that penetrated the inner third of the dentine, devoid of clinical manifestations of irreversible pulpitis or radiographic indications of periapical or furcation pathology. Only molars that could be restored and had confirmed pulpal vitality were included. Teeth that had spontaneous pain, swelling, sinus tract formation, percussion tenderness, pathological mobility, internal or external resorption, or any radiographic periapical changes were not included. Primary molars with fewer than twelve months until natural exfoliation and permanent molars with incomplete root development were also omitted. The sample size for the study was determined based on previously documented favourable pulpal responses of approximately 80-85% subsequent to SDF application. We figured out that we needed at least 28 teeth per group to get a statistically significant difference of 0.20, a confidence level of 0.05 and a power of 80%. After taking into account a drop-out rate of 10-15%, the final sample size was 70 teeth, including 35 primary molars and 35 permanent molars with deep carious lesions. All procedures were performed under stringent aseptic conditions. A clinical exam and baseline X-rays were done for each tooth to check the depth of the lesion and the health of the pulp. When it was possible, a rubber dam was used to keep teeth separate. When that wasn't possible, cotton rolls and high-volume suction were used instead. The removal of caries was done using a minimally invasive method called selective-caries- removal. The peripheral caries was completely removed until firm dentine was reached. The soft infected dentine that was on top of the pulpal wall was only partially removed to keep the pulp from being exposed. The deepest dentinal areas were left with hard, leathery dentine to keep the pulp alive. After the cavity was dug out, it was carefully rinsed and dried with cotton pellets, making sure not to dry out the dentine. A microbrush was used to apply a 38% SDF solution to the cavity floor for about one to three minutes. The solution only touched dentinal tissue and not gingival surfaces. To avoid irritating and staining the soft tissue, sterile cotton pellets were used to blot up the extra solution. After SDF was put on, the cavity was filled with either high-viscosity glass ionomer cement or resin-modified glass ionomer, depending on what was best for the patient. The occlusion was then adjusted as needed. Patients were asked to come back for clinical and radiographic tests every month, three months, six months and twelve months. During every visit, the tooth was checked for signs like pain that happened on its own, sensitivity, swelling, sinus tract formation and tenderness to percussion. Pulp vitality tests were compared to baseline responses to make sure that the pulp was still alive. Radiographic examination was conducted at baseline, six months and twelve months to evaluate the presence of periapical or furcal radiolucency, internal or external root resorption, widening of the periodontal ligament space and indications of arrested caries or tertiary dentine formation. A favourable pulpal response was characterised by sustained tooth vitality, the absence of symptoms and the lack of radiographic pathology. The emergence of spontaneous pain, loss of vitality, formation of a sinus tract, or radiographic indications of periapical or furcal pathology was classified as a treatment failure and addressed in accordance with established protocols, including pulpotomy or root canal therapy. We put all the data we collected into a structured proforma and used the right tests to analyse it statistically. For categorical variables, we used the chi-square or Fisher's exact test and for overall success rates, we used descriptive statistics. A p-value below 0.05 was deemed statistically significant. The methodology guaranteed standardised clinical protocols, dependable follow-up and impartial evaluation of pulpal response in both primary and permanent molars subjected to SDF treatment.

## Results:

A total of 70 teeth (35 primary molars and 35 permanent molars) were evaluated over a 12-month period. All participants completed the baseline, 1-month, 3-month and 6-month follow-ups, while 67 teeth completed the 12-month follow-up. The overall distribution of favourable and unfavourable pulpal responses across primary and permanent molars is presented in (Table 1). At the 1-month evaluation, 91.4% of primary molars and 94.2% of permanent molars remained asymptomatic with normal vitality responses. Only 3 teeth (1 primary, 2 permanent) reported mild transient postoperative sensitivity that subsided without intervention. No teeth showed radiographic abnormalities at this stage. By the 3-month follow-up, the favourable response rate was 88.6% in primary molars and 91.4% in permanent molars. Three teeth developed persistent symptoms and were categorized as failures. Radiographically, two primary molars exhibited early widening of the periodontal ligament space (Table 2). At 6 months, 82.9% of primary molars and 88.6% of permanent molars continued to show favourable outcomes (Table 1). Clinical failures were primarily due to spontaneous pain or development of a sinus tract. Radiographically, four teeth (three primary, one permanent) demonstrated either furcal radiolucency or initial root resorption. At the 12-month evaluation, the overall success rate was 80.0% in primary molars and 85.7% in permanent molars (Table 1). Permanent molars showed a slightly higher rate of normal vitality response (85.7%) compared with primary molars (80.0%), although the difference was not statistically significant (p > 0.05). Reduced but positive vitality responses were observed in 11.4% of primary molars and 8.6% of permanent molars, while absence of response indicating pulpal necrosis was noted in 8.6% and 5.7% of teeth, respectively (Table 3).

## Discussion: 

Silver diamine fluoride has gained attention as an effective non-invasive material for managing deep carious lesions. Its ability to arrest caries and preserve tooth structure supports the principles of minimally invasive dentistry [10]. The antimicrobial activity of SDF plays a crucial role in controlling cariogenic bacteria within dentinal lesions. Silver ions disrupt bacterial cell walls and metabolic pathways, thereby reducing microbial activity [11]. Fluoride present in SDF enhances remineralization of demineralized dentin and enamel. This process increases resistance of the tooth structure to further acid attack [12]. Previous clinical studies have reported that SDF can successfully arrest caries progression in both primary and permanent teeth. Its effectiveness has made it a valuable alternative in situations where conventional restorative treatment is difficult [13]. Another important aspect is the interaction of SDF with dentinal tubules and the dentin-pulp complex. Studies have shown that SDF can occlude dentinal tubules and reduce dentin permeability [14]. This tubule occlusion may help protect the pulp by limiting bacterial penetration and reducing external irritants reaching the pulpal tissue. Such effects contribute to maintaining pulpal vitality in deep lesions [15]. Structural changes in dentin following SDF application have also been reported. Formation of silver-containing compounds and fluorohydroxyapatite strengthens the affected dentin [16]. Experimental investigations have further suggested that SDF may influence the biological response of pulpal cells. These responses may include reduced inflammatory activity and stimulation of protective dentin formation [17]. Clinical studies evaluating pulpal outcomes have generally shown favorable results when SDF is applied to deep carious lesions without direct pulp exposure. The pulp tissue often remains vital with minimal inflammatory changes [18]. However, the effectiveness and biological response may depend on lesion depth, application technique and tooth type. Careful case selection remains essential to achieve optimal outcomes [19]. Some investigations have also highlighted the importance of monitoring treated teeth over time. Long-term evaluation helps confirm continued caries arrest and pulpal health [20]. Earlier histological studies have indicated that conservative management of deep caries can maintain pulp vitality when bacterial load is controlled. This supports the biological rationale behind SDF therapy [21]. Recent systematic reviews have emphasized the growing clinical interest in SDF as a preventive and therapeutic agent. Its ease of application and cost-effectiveness make it suitable for community dental programs [22]. Furthermore, laboratory studies have demonstrated that SDF may influence dentin mineral content and microstructure. These changes contribute to increased hardness and resistance of carious dentin [23]. Overall, the findings support the potential of SDF as a useful material for managing deep carious lesions while preserving pulpal health. Further clinical and histological studies are recommended to better understand its long-term pulpal response.

## Conclusion:

We show that 38% silver diamine fluoride is effective in maintaining pulpal vitality in the majority of deep carious primary and permanent molars. A favourable clinical and radiographic response was observed over 12 months, with relatively few failures. Overall, SDF appears to be a safe, minimally invasive option for managing deep caries while supporting pulp preservation.

## Advancement to knowledge:

This study provides additional evidence regarding the biological response of pulpal tissues following the application of silver diamine fluoride (SDF) in deep carious lesions. It contributes to existing literature by evaluating the pulpal response in both primary and permanent molars under *in vivo* conditions. The findings enhance understanding of the safety and effectiveness of SDF as a minimally invasive treatment for deep caries management. Furthermore, the study supports the potential role of SDF in preserving pulp vitality while arresting caries progression.

## Figures and Tables

**Table 1:** Overall clinical success (favourable pulpal response) at each follow-up interval

**Follow-up Interval**	**Primary Molars (n=35)**	**Permanent Molars (n=35) **	**p-value**
1 month	32 (91.4%)	33 (94.2%)	0.64
3 months	31 (88.6%)	32 (91.4%)	0.68
6 months	29 (82.9%)	31 (88.6%)	0.47
12 months	28 (80.0%)	30 (85.7%)	0.53

**Table 2:** Radiographic findings at follow-up intervals

**Finding**	**1 Month**	**3 Months**	**6 Months**	**12 Months**
No radiographic	100%	94.30%	88.60%	85.70%
abnormality				
Widened PDL space	0%	2.80%	4.30%	5.70%
Furcal/periapical	0%	2.80%	5.70%	7.10%
radiolucency				
Initial external resorption	0%	0%	1.40%	1.40%

**Table 3:** Vitality test responses at 12 months

**Response Type**	**Primary Molars (n=35)**	**Permanent Molars (n=35)**
Normal response	28 (80.0%)	30 (85.7%)
Reduced but positive response	4 (11.4%)	3 (8.6%)
No response (necrosis)	3 (8.6%)	2 (5.7%)
